# Assessing volumetric brain differences in migraine and depression patients: a UK Biobank study

**DOI:** 10.1186/s12883-023-03336-x

**Published:** 2023-07-28

**Authors:** Oreste Affatato, Amelia D. Dahlén, Gull Rukh, Helgi B. Schiöth, Jessica Mwinyi

**Affiliations:** 1 Department of Surgical Science, Group of Functional Pharmacology and Neuroscience, Uppsala University, Uppsala, Sweden; 2 Uppsala University’s Centre for Women’s Mental Health During the Reproductive Lifespan - WoMHeR, University of Uppsala, Uppsala, Sweden

**Keywords:** Migraine, Depression, Structural brain MRI, UK Biobank

## Abstract

**Background:**

Migraine and depression are two of the most common and debilitating conditions. From a clinical perspective, they are mostly prevalent in women and manifest a partial overlapping symptomatology. Despite the high level of comorbidity, previous studies hardly investigated possible common patterns in brain volumetric differences compared to healthy subjects. Therefore, the current study investigates and compares the volumetric difference patterns in sub-cortical regions between participants with migraine or depression in comparison to healthy controls.

**Methods:**

The study included data from 43 930 participants of the large UK Biobank cohort. Using official ICD10 diagnosis, we selected 712 participants with migraine, 1 853 with depression and 23 942 healthy controls. We estimated mean volumetric difference between the groups for the different sub-cortical brain regions using generalized linear regression models, conditioning the model within the levels of BMI, age, sex, ethnical background, diastolic blood pressure, current tobacco smoking, alcohol intake frequency, Assessment Centre, Indices of Multiple Deprivation, comorbidities and total brain volume.

**Results:**

We detected larger overall volume of the caudate (mean difference: 66, 95% CI [-3, 135]) and of the thalamus (mean difference: 103 mm^3^, 95% CI [-2, 208]) in migraineurs than healthy controls. We also observed that individuals with depression appear to have also larger overall (mean difference: 47 mm^3^, 95% CI [-7, 100]) and gray matter (mean difference: 49 mm^3^, 95% CI [2, 95]) putamen volumes than healthy controls, as well as larger amygdala volume (mean difference: 17 mm^3^, 95% CI [-7, 40]).

**Conclusion:**

Migraineurs manifested larger overall volumes at the level of the nucleus caudate and of the thalamus, which might imply abnormal pain modulation and increased migraine susceptibility. Larger amygdala and putamen volumes in participants with depression than controls might be due to increased neuronal activity in these regions.

## Introduction

Migraine is a debilitating neurological disorder characterized by severe and recurrent headaches, and one of the leading causes of disability worldwide [1]. Migraine is also characterized by high degree of comorbidity with a variety of psychiatric, neurologic, vascular, and cardiac conditions [2, 3]. This interconnection is of relevance in the clinical practice, as it might influence the efficacy of treatments. Regarding psychiatric conditions, epidemiological studies have shown that migraineurs have an increased risk of developing depression, anxiety and suicidal behavior when compared to non-migraineurs [2]. More specifically, major depressive disorder (MDD) has shown to be the most frequent psychiatric diagnosis among patients with migraine, especially in chronic or with aura subtypes [3–5]. The relation between the two disorders is bidirectional, i.e. having one disorder significantly increases the risk of manifesting the other one as well [6, 7]. This epidemiological evidence of a relationship between the two health conditions is not yet fully understood but might be the result of a partly overlapping pathophysiology that connects pathological pathways of migraine and depression.

With regard to clinical presentation, migraine and depression affect mostly women and are characterized by heterogeneity and partial overlap of symptoms [1, 8]. Their symptomatology is also shared with similar disorders, e.g. generalized anxiety disorder, and this may complicate the diagnosis and treatment selection [9]. The overlapping symptomatology and the high degree of comorbidity of migraine and depression supports the hypothesis that they might be the results of a common pathophysiological pathway. In this sense, increasing attention has been devoted in recent years to structural and functional brain imaging techniques [10, 11]. In the case of MDD, structural magnetic resonance imaging (MRI) studies have shown lower brain volumes in regions involved in emotional processing including amygdala and cingulate cortex, but also in other areas such as frontal cortex, orbitofrontal cortex, hippocampus, striatum and cerebellum [10, 12–14]. In the case of migraine, the structural alterations of the brain are related to regions implicated in pain experience and in visual and motion processing [11, 15]. Studies have shown that migraineurs manifested lower volumes in the bilateral insula, frontal/prefrontal, temporal, parietal and occipital cortices, as well as the anterior cingulate cortex, basal ganglia and cerebellum [16, 17]. These studies showed that subjects with migraine and depression manifest overall different volumetric patterns in the brain regions analyzed (with few exceptions, such the anterior cingulate cortex and the amygdala). This could be due to the different choice of parameters used for the MRI scan. Moreover, the aforementioned studies are generally characterized by small sample sizes. To overcome these limitations, we used large-scale UK Biobank data to perform study with a larger sample size. Another important feature of the UK Biobank cohort is that for the MRI scans it was adopted a unique methodology in all the Assessment Centres. The comprehensive dataset of this cohort grants the possibility of a more in-depth analysis of the structural changes in the various brain regions and to reduce the confounding level adjusting our statistical model using many important variables.

The purpose of the current study is to provide a more comprehensive and in-depth set of measurements of subcortical volumetric changes in gray and white matter in subjects with migraine or depression in comparison to healthy controls to elucidate common and different morphological features of these two disorders.

## Materials and methods

### Cohort

Data were provided by the UK Biobank, a biorepository based on a large population cohort with approximately half a million participants from the United Kingdom. This database contains in-depth genetic information as well as comprehensive health and lifestyle data accessible to approved research. The UK Biobank study principal aim is to promote research on a wide range of important health conditions. Ethical approval for the UK Biobank study was granted by the North-West Multicenter Research Ethics Committee (permission UKB 57519). The Regional Ethics Committee of Uppsala (Sweden) approved the use of UK Biobank data for the present study (2017/198).

### Primary outcome variables

UK Biobank provided several variables to investigate subcortical volumes of different brain regions. In particular, we considered gray matter volumes of thalamus, caudate, putamen, pallidum, hippocampus and amygdala. The volumes were obtained by means of T1 structural brain imaging using FAST tool for segmentation/registration [18]. We also considered overall volumes (gray and white matter) of thalamus, caudate, putamen, pallidum, hippocampus, amygdala and nucleus accumbens. These volumes were obtained by means of T1 structural brain imaging using FIRST tool for segmentation/registration [19]. FAST and FIRST segmentation tools differ in several features and working assumptions and we adopted both of them since the UK Biobank researchers performed FIRST-based analyses only for the overall subcortical regions and the FAST-based analyses only for measuring the gray matter volumes of the same areas. All the volumes are expressed in mm^3^. Brain images have been acquired using 3 T Siemens Skyra (software platform VD13), with standard Siemens 32-channel RF receive head coil [20]. Complete information regarding the neuroimaging process, from machinery used to protocols, can be retrieved here at the following link: https://biobank.ctsu.ox.ac.uk/crystal/crystal/docs/brain_mri.pdf.

### Covariates

The main predictors of our analyses are the diagnosis of migraine and depression. To identify the cases (migraine and depression) and the controls we used the variable “Diagnoses – ICD10”, which contains the information of every diagnosis from the inpatient hospital registries for each participant. We identified the migraine cases using all the diagnosis of the category G43 and similarly depression cases the diagnosis under the categories F32 and F33.

To reduce the level of confounding we included in our statistical model many comorbidities and health-conditions. In particular, we considered: viral and bacterial infections of the nervous system (A80, A81—A85, A87, A88, G00, G02—G06), diabetes (E10—E14), diseases of the nervous system (G10—G13, G21, G23—G25, G30—G32, G36, G37), mental and behavioral disorders due to psychoactive substances (F10—F19), psychiatric, mental and behavioral disorders (F00—F02, F05—F07, F20, F22, F23, F25, F30, F31, F34, F38, F40—F45, F48, F50, F53, F54, F62, F63, F68, F99), developmental disorders (F70—F73, F78—F81, F84, F88, F89), epilepsy and sleep disorders (G40, G41, G47, F51), muscle disorders (G56, G71—G73, G80—G83), headaches other than migraine (G44), neuropathies (G50—G55, G57—G63, G70, G90), brain and spine malformations/abnormalities (G91, G93 117 - G97, G99, Q00, Q01, Q03, Q07), cerebrovascular diseases (I60—I63, I65—I69, G45, G46), head and spine injuries and fractures (S001, S007—S010, S01, S02, S020—S024, S026—S029, S04, S06—S09), cardiovascular diseases (I00—I02, I05—I13, I15, I20—I28, I30—I37, I39, I40—I52, I70—I74, I77—I80, I82—I89, I95, I97, I98) and brain cancers (C70—C75, D32, D33, D43).

We considered also important biological covariates such as sex, body mass index (BMI), diastolic blood pressure, age, ethnic background, current tobacco smoking, and alcohol intake frequency. Moreover, we included sociodemographic variables such as Assessment Center and the indices of multiple deprivation (IMD). The Assessment Center variable contains information on which center was visited by each participant. The IMD is a measurement of poverty in small areas, an indicator widely used in the United Kingdom. The IMD comprise several domains of deprivation such as income, health, employment, crime, education barriers to housing and services, and living environment.

### Statistical methods

We used descriptive statistics to summarize the general features of the two study arms. Table 1 displays the results. We also calculated mean, median, standard deviation (SD), and interquartile range (IQR) for all the subcortical regions. In Table 2 we reported the results for the overall volumes, while in Table 3 we reported the results for the gray matter volumes.

**Table 1:** Descriptive statistics of main sociodemographic factors

	Migraine *N* = 712	Depression *N* = 1 853	Controls *N* = 43 930
*Sex*			
Women	527 (74%)	1 180 (64%)	22 881 (52%)
Men	185 (26%)	673 (36%)	21 049 (48%)
*Age* (mean ± SD)	63 ± 8	63 ± 8	64 ± 8
*BMI* (mean ± SD)	27 ± 5	28 ± 5	26 ± 4
*Ethnic background*			
British	644 (90%)	1 701 (92%)	39 962 (91%)
Irish	18 (2%)	54 (3%)	1 122 (3%)
Any other white background	25 (4%)	54 (3%)	1 364 (3%)
Others	25 (4%)	44 (2%)	1 482 (3%)
IMD (median, IQR)	12 [7, 21]	13 [8, 25]	11 [7, 20]
*Current tobacco smoking*			
Yes, on most or all days	14 (2%)	89 (5%)	828 (2%)
Only occasionally	10 (1%)	49 (3%)	569 (1%)
No	682 (97%)	1 696 (92%)	42 223 (97%)
Prefer not to answer	0 (0%)	0 (0%)	8 (0%)
*Alcohol intake frequency*			
Daily or almost daily	60 (8%)	295 (16%)	7 389 (17%)
Three or four times a week	136 (20%)	301 (16%)	12 426 (28%)
Once or twice a week	174 (25%)	241 (12%)	11 596 (26%)
One or three times a month	97 (13%)	400 (21%)	5 009 (12%)
Special occasions only	135 (20%)	377 (19%)	4 447 (11%)
Never	104 (14%)	295 (16%)	2 745 (6%)
Prefer not to answer	0 (0%)	1 (0%)	16 (0%)

**Table 2:** Descriptive statistics for the overall volumes (expressed in mm^3^) of the subcortical regions under study displayed for cases and controls (FIRST segmentation tool). We calculated mean, median, standard deviation (SD), and interquartile range (IQR)

Brain region	Migraine (*N* = 712)	Depression (*N* = 1 853)	Controls (*N* = 43 930)
Thalamus	Mean = 15 064, Median = 15 035 SD = 1 377 IQR = [14 131, 15 990]	Mean = 15 076, Median = 15 026 SD = 1 558 IQR = [14 067, 15 990]	Mean = 15 264, Median = 15 193 SD = 1 496 IQR = [14 238, 16 206]
Caudate	Mean = 6 843, Median = 6 768 SD = 841 IQR = [6 262, 7 382]	Mean = 6 891, Median = 6 855 SD = 877 IQR = [6 302, 7 417]	Mean = 6 927, Median = 6 882 SD = 846 IQR = [6 346, 7 454]
Putamen	Mean = 9 364, Median = 9 325 SD = 1 083 IQR = [8 600, 10 100]	Mean = 9 471, Median = 9 396 SD = 1 225 IQR = [8 635, 10 244]	Mean = 9 552, Median = 9 499 SD = 1 165 IQR = [8 753, 10 296]
Pallidum	Mean = 3 469, Median = 3 420 SD = 438 IQR = [3 171, 3 737]	Mean = 3 490, Median = 3 460 SD = 470 IQR = [3 180, 3 760]	Mean = 3 547, Median = 3 509 SD = 468 IQR = [3 237, 3 812]
Hippocampus	Mean = 7 535, Median = 7 546 SD = 847 IQR = [7 012, 8 044]	Mean = 7 551, Median = 7 531 SD = 920 IQR = [6 967, 8 143]	Mean = 7 639, Median = 7 640 SD = 895 IQR = [7 069, 8 207]
Amygdala	Mean = 2 400, Median = 2 392 SD = 414 IQR = [2 103, 2 688]	Mean = 2 467, Median = 2 461 SD = 423 IQR = [2 179, 2 735]	Mean = 2 489, Median = 2 478 SD = 438 IQR = [2 192, 2 772]
Nucleus Accumbens	Mean = 864, Median = 873 SD = 205 IQR = [734, 991]	Mean = 854, Median = 844 SD = 218 IQR = [710, 996]	Mean = 871, Median = 869 SD = 211 IQR = [727, 1012]

**Table 3:** Descriptive statistics for the gray matter volumes (expressed in mm^3^) of the subcortical regions under study displayed for cases and controls (FAST segmentation method). We calculated mean, median, standard deviation (SD), and interquartile range (IQR)

Brain region	Migraine (*N* = 712)	Depression (*N* = 1 853)	Controls (*N* = 43 930)
Thalamus	Mean = 5 458, Median = 5 429 SD = 548 IQR = [5 095, 5 833]	Mean = 5 510, Median = 5 487 SD = 567 IQR = [5 120, 5 835]	Mean = 5 526, Median = 5 494 SD = 579 IQR = [5 142, 5 867]
Caudate	Mean = 6 279, Median = 5 970 SD = 1 430 IQR = [5 386, 6 737]	Mean = 6 400, Median = 6 062 SD = 1 579 IQR = [5 439, 6 905]	Mean = 6 334, Median = 6 049 SD = 1 461 IQR = [5 417, 6 860]
Putamen	Mean = 3 881, Median = 3 815 SD = 866 IQR = [3 306, 4 355]	Mean = 3 908, Median = 3 847 SD = 914 IQR = [3 315, 4 397]	Mean = 3 930, Median = 3 866 SD = 860 IQR = [3 358, 4 427]
Pallidum	Mean = 96, Median = 79 SD = 69 IQR = [58, 107]	Mean = 100, Median = 81 SD = 85 IQR = [60, 112]	Mean = 103, Median = 84 SD = 82 IQR = [62, 115]
Hippocampus	Mean = 8 374, Median = 8 338 SD = 8 22 IQR = [7 852, 8 917]	Mean = 8 418, Median = 8 367 SD = 862 IQR = [7 847, 8 946]	Mean = 8 535, Median = 8 505 SD = 837 IQR = [7 967, 9 060]
Amygdala	Mean = 3 828, Median = 3 808 SD = 482 IQR = [3 526, 4 140]	Mean = 3 811, Median = 3 804 SD = 516 IQR = [3 475, 4 144]	Mean = 3 906, Median = 3 895 SD = 488 IQR = [3 587, 4 222]

**Fig. 1:**
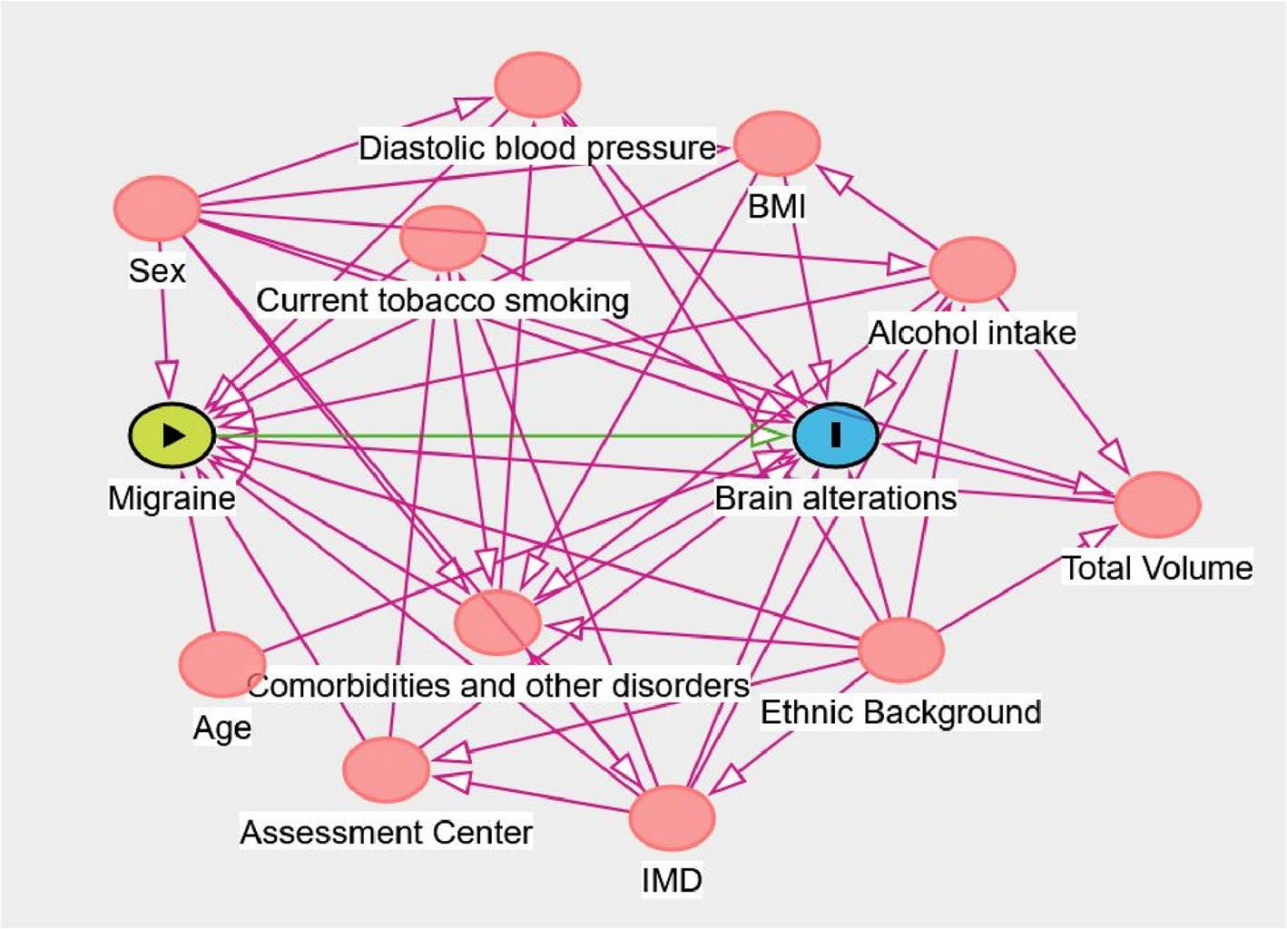
Causal directed acyclic graph (cDAG) for our causal model. In red are represented all the paths that introduce confounding. The cDAG was drawn using DAGitty v3.0

The primary aim of this study is to estimate the volumetric differences in several subcortical brain regions between cases (migraine or depression) and healthy controls. To do this we used multiple linear models, where the outcome variable Y is the volume (expressed in mm^3^) of the target brain region. We conditioned the model within the levels of several covariates to reduce the bias due to confounding. For the choice of the appropriate set of predictors we used causal directed acyclic graphs (cDAGs). The cDAG summarizing our model assumptions is displayed in Fig. 1. In particular, we considered as relevant predictors for our model body mass index (BMI) [21–23], age [24–26], sex, ethnical background, diastolic blood pressure, current tobacco smoking, alcohol intake frequency, Assessment Centre, IMD, comorbidities and major health-related conditions (i.e. all the health conditions we mentioned in the *Covariates* sub-section) and total brain volume (grey and white matter, normalized for head size).

UK Biobank provided two separate values for each region, the left and the right part. We summed these two values, so Y represents the overall volume of that region. X_diag_ is the categorical variable which represents the diagnosis (migraine or depression and healthy controls). This is the equation we interpolated with our data

$$Y= \alpha +{\beta }_{diag}{X}_{diag}+{\beta }_{sex}{X}_{sex}+{\beta }_{age}{X}_{age}+{\beta }_{BMI}{X}_{BMI}+{\beta }_{Alch}{X}_{Alch}+{\beta }_{Smok}{X}_{Smok}+{\beta }_{DBP}{X}_{DBP}+{\beta }_{IMD}{X}_{IMD}+{\beta }_{AssC}{X}_{AssC}+{\beta }_{Comorb}{X}_{Comorb}+{\beta }_{ethn}{X}_{ethn}+{\beta }_{brain}{X}_{brain}+ \varepsilon$$

We fitted this model with our data to obtain an estimation of the β_diag_ parameter and the 95% Confidence Interval (95% CI). We did not correct the confidence level for the multiplicity problem, as it would decrease the precision and increase the type II error rate [27]. For each interpolation, X_diag_ was a dichotomous variable, and the control group was considered the reference group (with assigned value zero, while the other group was assigned value one). Therefore, the β_diag_ parameter represents the difference between the mean volumes in the two groups.

We focus on point and interval estimation. Statistical inference is therefore based on estimation, which is better suited for the task rather than the less informative hypothesis testing [28–31]. For this reason, no test of hypothesis has been performed and therefore no significance threshold was established and no *p*-values were reported, as possibly misleading [32–35]. Furthermore, in order to have a better understanding of the relative magnitude of volume differences between the groups we complemented the estimations of the mean difference and the relative standard error (SE), with the Cohen’s d [36]. We used the formula:

$$d= \frac{t({n}_{1}+ {n}_{2})}{\sqrt{{n}_{1}{n}_{2}} \sqrt{df}}$$

where t is the difference between the means (${\beta }_{diag}$) divided by the corresponding standard error, n_1_ and n_2_ are the sample sizes of cases and controls respectively and df are the degrees of freedom for the t value, i.e. df = n_1_ + n_2_ – 2. We referred to the usual classification of the d values, as stated by Cohen: small (d = 0.2), medium (d = 0.5) and big effect (d = 0.8) [36–38].

To give a quantitative measure of the relative precision of our estimations we also calculated the relative error

$${\varepsilon }_{r}= \frac{SE}{{\beta }_{diag}} \times 100\%$$

All statistical analyses have been conducted using R and RStudio (R version 4.1.1 [64 bit], RStudio version 1.4.1106). The complete script used for data curation and the statistical analyses is available on GitHub at the following link: https://github.com/OresteAffatato/Migraine_Depression_MRI_project.

## Results

After the exclusion of the participants without MRI brain scan, we obtained a final sample that comprises 712 individuals with migraine, 1 853 with depression and 43 930 controls. In Table 1 are summarized main sociodemographic features of the sample.

Tables 2 and 3 display the main descriptive statistics of the volumes of the different subcortical regions. In Table 2 we reported the overall volumes (gray + white matter) statistics, based on the FIRST segmentation tool, while in Table 3 we reported the descriptive statistics of the gray matter volumes of the same subcortical regions (except nucleus accumbens), based on the FAST segmentation method.

We can observe from the descriptive statistics displayed in Tables 2 and 3 some general features. In all cases, mean and median are close to each other, therefore the distributions of the volumes of each region and each group are symmetric. The standard deviations (SD) are generally quite large, as the IQRs. Summarizing these facts, we can conclude that the volumetric distributions of each region between cases and control are extensively overlapping.

### Differences in overall subcortical volumes

Table 4 displays the mean differences of overall subcortical volumes (gray + white matter) between migraineurs and controls. The strongest effects, as measured in absolute (${\beta }_{diag}$) and relative (Cohen’s d) terms, are at the thalamic (mean difference: 103 mm^3^, 95% CI [-2, 208]) and caudate (mean difference: 66 mm^3^, 95% CI [-3, 135]) levels, were migraineurs appear to have larger volumes than controls.

**Table 4:** Mean volume difference between migraine cases and controls. FIRST segmentation method. All volumes are expressed in mm^3^

Brain region	Mean ± SE	${\varepsilon }_{r}$	95% Confidence Interval	Cohen’s d
Thalamus	103 ± 55	53%	[-2, 208]	0.08
Caudate	66 ± 35	53%	[-3, 135]	0.08
Putamen	45 ± 43	96%	[-38, 129]	0.04
Pallidum	11 ± 19	173%	[-27, 49]	0.03
Hippocampus	-9 ± 36	400%	[-79, 61]	-0.01
Amygdala	-8 ± 18	225%	[-45, 27]	-0.02
Nucleus accumbens	2 ± 8	400%	[-14, 17]	0.01

Table 5 shows mean differences in overall volumes between depression cases and healthy controls. Notably, individuals with depression appear to have larger volumes than controls at the level of the putamen (mean difference: 47 mm^3^, 95% CI [-7, 100]) and of the amygdala (mean difference: 17 mm^3^, 95% CI [-7, 40]).

**Table 5:** Mean volume difference between depression cases and controls. FIRST segmentation method. All volumes are expressed in mm^3^

Brain region	Mean ± SE	${\varepsilon }_{r}$	95% Confidence Interval	Cohen’s d
Thalamus	-10 ± 34	340%	[-77, 58]	-0.01
Caudate	17 ± 22	130%	[-27, 61]	0.03
Putamen	47 ± 27	57%	[-7, 100]	0.07
Pallidum	2 ± 12	600%	[-22, 26]	0.01
Hippocampus	-17 ± 23	135%	[-61, 28]	-0.03
Amygdala	17 ± 12	71%	[-7, 40]	0.06
Nucleus accumbens	-2 ± 5	250%	[-12, 8]	-0.02

**Fig. 2:**
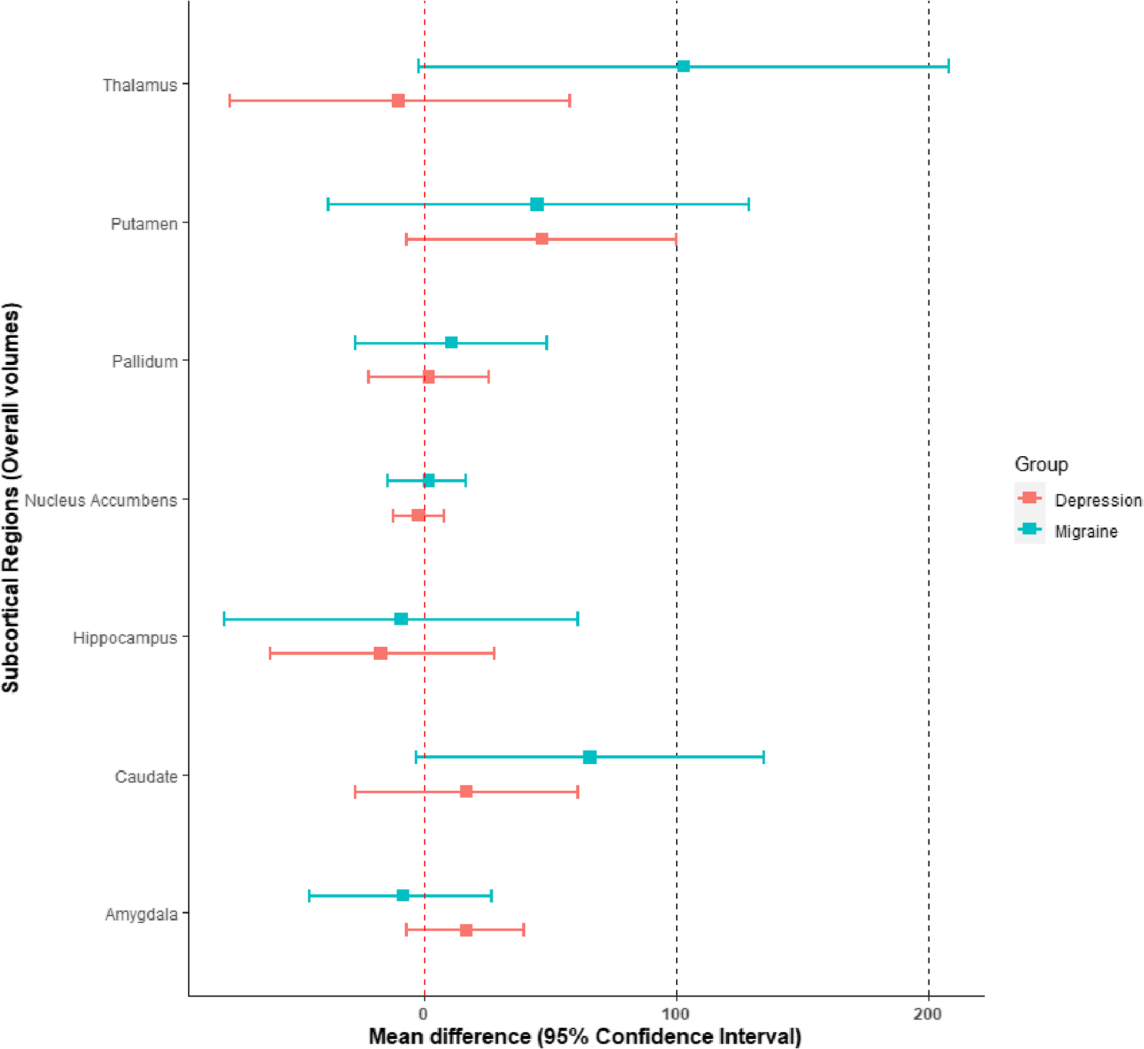
Forest plot displaying the volumetric differences (means and 95% CIs) for migraine and depression cases

In Fig. 2 are portrayed all the results for the overall volumes. The estimates are generally of small magnitude and affected by significant low precision. At the level of the pallidum and the nucleus accumbens, both individuals with migraine and depression do not appear to differ from the controls. At the level of the putamen both types of cases seem to have larger volumes than controls.

### Differences in gray matter subcortical volumes

In Table 6 are reported the results for migraine cases. The effect sizes are generally very small and characterized by low precision.

**Table 6:** Mean gray matter volume difference between migraine and controls. FAST segmentation method. All volumes are expressed in mm^3^

Brain region	Mean ± SE	${\varepsilon }_{r}$	95% Confidence Interval	Cohen’s d
Thalamus	8 ± 24	300%	[-40, 56]	0.01
Caudate	-13 ± 62	477%	[-134, 108]	-0.01
Putamen	45 ± 37	82%	[-28, 118]	0.05
Pallidum	-1 ± 3	300%	[-8, 6]	-0.01
Hippocampus	-10 ± 33	330%	[-75, 55]	-0.01
Amygdala	8 ± 18	225%	[-28, 44]	0.02

Table 7 show the results for depression cases. Notably, individuals with depression appear to have larger gray matter volumes at putamen level (mean difference: 49 mm^3^, 95% CI [2, 95]). They also appear to have lower gray matter volumes at the level of the amygdala (mean difference: -21 mm^3^, 95% CI [-44, 2]).

**Table 7:** Mean gray matter volume difference between depression and controls. FAST segmentation method. All volumes are expressed in mm^3^

Brain region	Mean ± SE	${\varepsilon }_{r}$	95% Confidence Interval	Cohen’s d
Thalamus	5 ± 16	320%	[-25, 36]	0.02
Caudate	15 ± 40	267%	[-63, 92]	0.02
Putamen	49 ± 24	49%	[2, 95]	0.09
Pallidum	-1 ± 2	200%	[-6, 3]	-0.02
Hippocampus	-27 ± 21	78%	[-69, 15]	-0.06
Amygdala	-21 ± 12	57%	[-44, 2]	-0.08

**Fig. 3:**
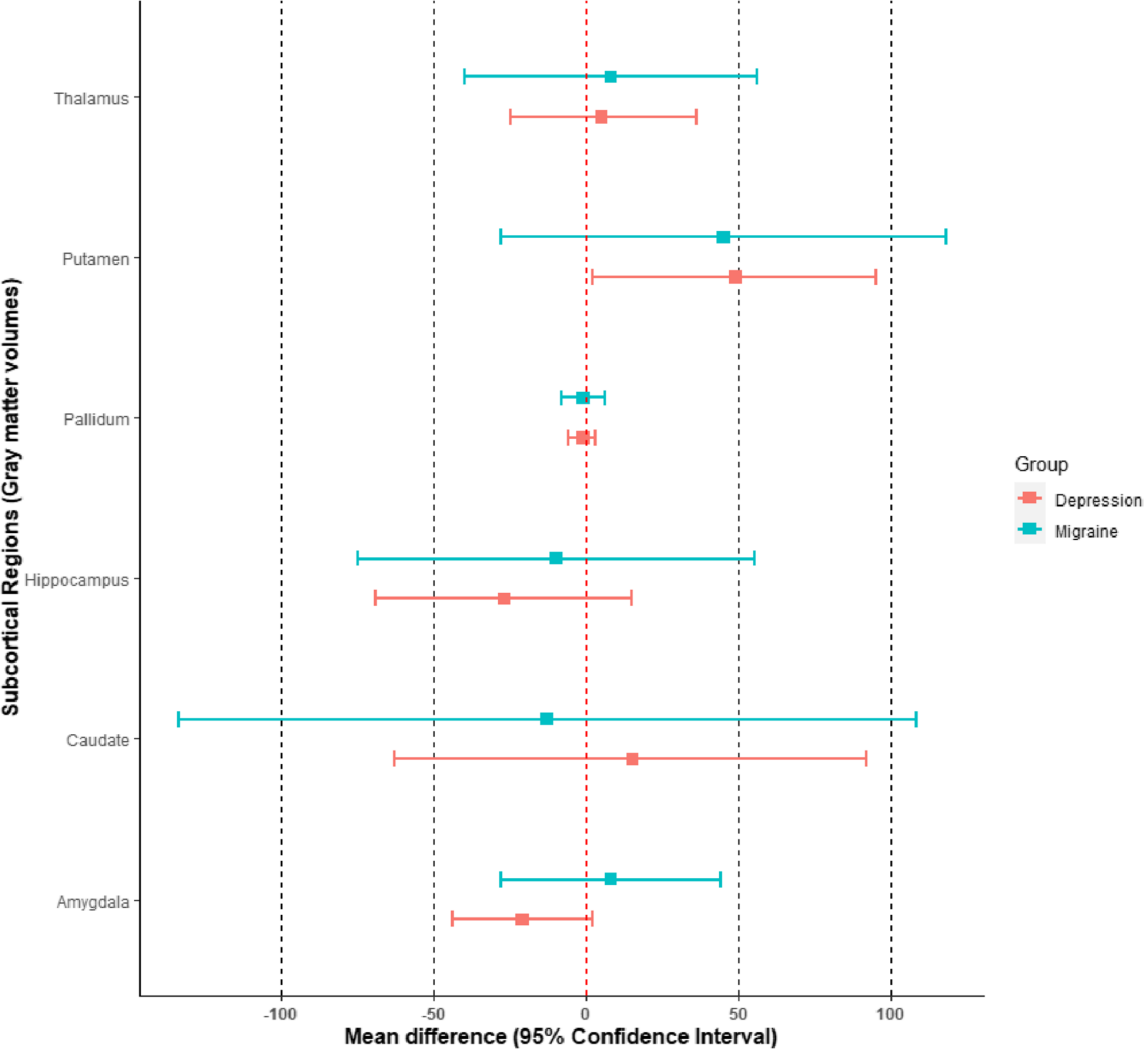
Forest plot displaying the gray matter volumetric differences (means and 95% CIs) for migraine and depression cases

Figure 3 displays the forest plot of the results for the gray matter volumes. As in the previous case, the estimates are generally characterized by small effect size and low precision. Notably, also at the level of gray matter both migraine and depression cases appear to have no significant pallidum volumetric difference from controls.

## Discussion

To the best of our knowledge, this is the first study that addresses associations between migraine and depression diagnosis and subcortical volumetric differences using data from a large population-based cohort. Notably, participants with migraine manifested larger overall volume of the caudate (mean difference: 66 mm^3^, 95% CI [-3, 135]) than healthy controls. The nucleus caudate is known to have important functional connections with other brain regions which are likely to be integral in migraine pathophysiology [39]. The caudate has also been shown to manifest anti-nociceptive functions and to play a role in pain modulation in connection with the periaqueductal gray matter [40]. Therefore, abnormal activity at the level of the nucleus caudate might imply pain regulation dysfunction which could in turn increase migraine susceptibility. It has been also shown that the cortical spreading depression, a depolarization wave that is associated with migraine, inhibits the neuronal activity in the caudate [39, 41]. This reduced activity might imply disruption of pain regulation and therefore lead to migraine pain. Moreover, we found larger overall volume of the thalamus (mean difference: 103 mm^3^, 95% CI [-2, 208]) than controls. The thalamus is known to play an important role in migraine pathophysiology and therefore the larger thalamic volume could be due to the increased activity of this region in subjects with migraine [1, 42].

We also observed that subjects with depressive symptoms manifested a larger overall amygdala volume (mean difference: 17 mm^3^, 95% CI [-7, 40]) than healthy controls. This phenotype might reflect increased activity of the neurons in this brain region. Other studies support the hypothesis that the hyper-activity of the amygdala increases the risk of developing depressive symptoms and related comorbidities [43]. Increased amygdala activity has been observed in people diagnosed with general internalizing disorders [44, 45]. Moreover, it has been shown reduced amygdalar reactivity after administration of effective treatment for depression and anxiety disorders [46–48]. These observations supports the hypothesis that an increased activity of the amygdala is associated with negative disposition, anxiety and internalizing symptoms and therefore to future development of depressive symptoms [43]. We also found lower gray matter amygdala volume (mean difference: -21 mm^3^, 95% CI [-44, 2]). The discrepancy between the overall and gray matter level could be explained by a compensation mechanism. Individuals with depression might have lower gray matter neurons and therefore the brain increases the white matter to compensate. The larger overall volume could be due over-compensation.

Individuals with depression appear to have also larger overall (mean difference: 47 mm^3^, 95% CI [-7, 100]) and gray matter (mean difference: 49 mm^3^, 95% CI [2, 92]) putamen volumes. The role of the putamen in depressive disorders has not been fully elucidated, and its pathophysiological involvement is currently under thorough investigation [49]. In particular, the putamen is known for playing an important role in motor control and movement disorders and for being an integral part of reward and learning circuits, which in turn play an important role in depressive conditions [50]. Previous literature has showed association between depressive symptoms and lower putamen volumes, in contrast with our findings. This could be due to different MRI and statistical analyses.

In general, we can observe that the volumetric differences were small. Moreover, even though in some cases the standard error was equal or larger than the effect sizes, the confidence intervals are also generally compatible with small effect sizes, in most of the cases. Our estimates were generally characterized by small precision, and therefore our findings should be generally treated with caution.

A strength of this study is that we provided a set of estimations of a wide variety of subcortical brain regions using a large cohort. To decrease the level of bias due to confounding we included in our model a wide set of important biological and sociodemographic predictors. Another important feature of this study is the analysis of the differences in gray and white matter volumes. Data from UK Biobank allowed us to assess not only brain volume differences among several subcortical regions, but also to address differences between white and gray matter volumes. Our study also provides many estimations that can be used in meta-analytical research to assess on stronger basis the actual direction of the associations for all the specific subcortical regions. A limitation of our study is related to the average age of the UK Biobank cohort. This sample comprises mainly older participants (mean age approximately 60 years), while migraine is known to be mostly prevalent in younger people [1]. Age does not only influence whether a participant is more likely to manifest a condition, but it has also an impact on brain structure and its functional connectivity [51]. These features pose limits on the generalization of our results. Another limitation is the cross-sectional nature of this study. We were not able to provide any causal trajectory between differences in subcortical structures and the presence of certain conditions. However, the patterns of association we found can be used to generate hypotheses that can lead to design studies assessing causal relationships. Another limitation from the UK Biobank is the sampling bias. The UK Biobank has an uncommonly low acceptance rate (around 6%) and this poses an important limitation on our research, given the presence of such a bias [52].

## Conclusion

This study provides estimations of subcortical volumes in a broad variety of brain regions as well as mean volumetric differences between subjects with migraine, depression and healthy controls. Migraineurs manifested larger overall volumes at the level of the nucleus caudate and of the thalamus. Abnormal activity in the caudate and the thalamus might imply abnormal pain modulation and increased migraine susceptibility. Subjects with depressive symptoms manifested larger amygdala and putamen overall volumes than controls, which might be due to increased activity in these regions. Migraine and depression are not likely to manifest similar patterns at the gross anatomy level in the main sub-cortical regions. Considering the large prevalence of migraine and depression in today’s society, mapping the neural signatures of the disorders will be critical for clarifying their causes.

## Data Availability

All data generated during this study are included in this paper and the data will be available made on request to corresponding author.

## Abbreviations

MDD: Major depressive disorder
MRI: Magnetic resonance imaging
IMD: Indices of Multiple Deprivation
BMI: Body mass index
SD: Standard deviation
SE: Standard error
CI: Confidence Interval
DAG: Directed acyclic graph
